# Measuring associations among British national identification, group norms and social distancing behaviour during the COVID‐19 pandemic: Testing a Social Identity Model of Behavioural Associations (SIMBA)

**DOI:** 10.1111/bjso.12862

**Published:** 2025-03-21

**Authors:** Emily A. Hughes, Joanne R. Smith

**Affiliations:** ^1^ University of Exeter Exeter UK

**Keywords:** balanced identity, COVID‐19, norms, social distancing, social identity

## Abstract

Social identification and group norms have been identified as key social psychological determinants of engagement in protective public health behaviours, such as social distancing, in the context of the COVID‐19 pandemic. Drawing upon both social identity and balanced identity theories, the research tests the utility of a Social Identity Model of Behavioural Associations (SIMBA)—which proposes reciprocal, interactive associations among self‐group, group‐behaviour and self‐behaviour concepts—for the measurement of British national identification, group norms and social distancing behaviour at two different points during the pandemic. An online study asked participants (Time 1 *N* = 151, Time 2 *N* = 136) to complete implicit and explicit (i.e. self‐report) measures both during and post‐lockdown. Results demonstrated associations to be relatively stable across time and found strong correlational confirmation that the strength of any one association in the SIMBA could be predicted by the interactive strength of the remaining two—both implicitly and explicitly. However, the strength of any one association, as measured post‐lockdown, was not predicted by the interaction between the change scores of the remaining two—suggesting that the constructs may not be long‐range predictors of one another. Findings are discussed in terms of the value of the SIMBA for the measurement and modification of novel, emergent group‐based associations.

## INTRODUCTION

As of January 2025, there have been more than 777 million confirmed cases of COVID‐19 worldwide, and over 7 million deaths (WHO, 2025)—rendering the COVID‐19 pandemic one of the greatest and most fatal health crises of recent times. The United Kingdom (UK) alone has witnessed over 25 million confirmed cases and 232,000 deaths with COVID‐19 on the death certificate (WHO, 2025). In the absence of an effective vaccine, mass behaviour change (e.g. in the form of social distancing, wearing face masks, regular hand washing) was fundamental as a first line of prevention to ‘flatten the curve’ of infections and ease strain on medical services. However, engagement with preventative health behaviours has been mixed. In the UK, for example, mask‐wearing was found to have the highest level of compliance, while compliance with social distancing was relatively low at points (Wright et al., 2021). Given that adherence to behavioural guidelines has implications for the trajectory of a pandemic—and that ‘social distancing’ in particular is a relatively novel, and therefore under‐researched, concept—there is a need to better understand this behaviour.

Some of the central social psychological factors that may be relevant to the prediction of social distancing behaviour in the context of COVID‐19 are social identification and group norms, and, in particular, the interaction between these two variables—which is widely recognized to be predictive of behaviour in a variety of health‐related domains such as regular exercise, sun protection (Terry & Hogg, 1996) and healthy eating (Åstrøm & Rise, 2001; Louis et al., 2007). Therefore, these variables are also likely to be highly explanatory in a public health context. It is also probable that relations among variables are reciprocal; while identity and norms may be predictive of behaviour, it is likely that norms and behaviour may be predictive of identity, and so on (Hughes & Smith, 2024). Accordingly, the current research tests the utility and validity of a Social Identity Model of Behavioural Associations (SIMBA) for the measurement of associations among British national identity, group norms and social distancing behaviour—both at cross‐sections of the pandemic and longitudinally over time—to examine associative strength and cognitive balance in the face of changing governmental guidelines and behavioural restrictions.

### Social norms and COVID‐19 preventative health behaviour

One important factor in predicting adherence to preventative public health behaviours is perceived social norms (Ruggeri et al., 2024; van Bavel et al., 2020). Social norms are ‘rules and standards that are understood by members of a group, and that guide and/or constrain social behaviour without the force of laws’ (Cialdini & Trost, 1998, p. 152). Observing what others do (i.e. descriptive norms) and what is approved of by others (i.e. injunctive norms) can provide individuals with useful information regarding the appropriate way to behave in a given situation, as behaviours that are common signal that they are also accurate, effective and that they elicit social approval (Eriksson et al., 2015; Mollen et al., 2010). This is particularly beneficial when the situational context is uncertain and ever‐changing, as is the case during a global pandemic. Here, individuals often look to social norms—in addition to guidance from authorities—to gain an understanding of how best to respond (Cialdini, 2001). Descriptive norms are likely to hold particular influence over behaviour (Elgaaied‐Gambier et al., 2018), as message recipients can easily and rapidly process information about how others behave and translate this into their own individual behaviour (Cialdini et al., 2006). In other words, the behaviour of those around us has consequences for our own behaviour, such as whether to comply with guidance and regulations prescribed by authorities.

The role of social norms in predicting individual behaviour is well documented in several behavioural contexts, including disease prevention. Evidence suggests that an individual's frequency of engaging in specific disease‐prevention behaviours is associated with their perceptions of their peers' frequency of engaging in those same behaviours (Dickie et al., 2018). Several correlational studies have also found positive associations among group norms, attitudes and behavioural intentions regarding vaccine uptake (e.g. Allen et al., 2009; Dillard, 2011; Nyhan et al., 2014). More specific to the COVID‐19 pandemic, a recent meta‐analysis of research investigating the relationship between constructs of the Theory of Planned Behaviour (TPB; Ajzen, 1991) and COVID‐19 preventative health behaviour more generally found subjective norms (i.e. perceived approval of a behaviour; Cialdini et al., 1990) to correlate strongly with both behavioural intentions and actual behaviour (Fischer & Karl, 2022). While norm effects are often weaker compared to attitudes and perceived behavioural control (PBC) when examining self‐oriented health (Armitage & Conner, 2001; McDermott et al., 2015; White et al., 2009), the pandemic presents a markedly different context in which health behaviours are only partially self‐oriented, and individual behaviour has wider‐reaching collective effects (Allcott et al., 2020; Prosser et al., 2020; Templeton et al., 2020). Under these circumstances, norms appear to be equally strong predictors of behaviour as attitudes and PBC; regarding actual behaviour, there were no significant differences between the three components of the TPB (Fischer & Karl, 2022). Moreover, the norm‐behaviour relationship was found to be stronger under conditions where subjective norms were more salient and participants on average felt that important others were strongly supportive of behaving in a protective way (Fischer & Karl, 2022). Most significantly, the association between descriptive norms and compliance with public health recommendations and prosocial behaviour recently emerged as one of the strongest empirical effects in social and behavioural science during COVID‐19 (Ruggeri et al., 2024)—thereby emphasizing the key role that norms play in the context of the pandemic, where individual behaviour is required to prevent collective harm.

### Social norms and social distancing behaviour

The concept of social distancing—in contrast to actions such as hand washing and getting vaccinated—represents a relatively novel behaviour. It is also one that individuals were required to assimilate to quickly, and comply with, at the beginning of the COVID‐19 pandemic prior to the development of pharmaceutical interventions. Consequently, identifying the key social psychological determinants of social distancing behaviour in particular—and the extent to which social norms may play a role in adherence to social distancing guidelines—became a critical focus of research. Recent research has continued to adopt a TPB framework to investigate the relationship between norms and social distancing behaviour. In a US sample, subjective norms were found to be a strong predictor of both behavioural intentions to engage in social distancing behaviour and actual social distancing behaviour through heightened perceived severity of COVID‐19 (Ang et al., 2021). Similarly, in both US and Australian samples, both subjective and moral norms (i.e., the extent to which a particular behaviour can be viewed as a moral responsibility/obligation) were found to be consistent predictors of behavioural intentions (Hagger et al., 2020). Intentions, action planning and habit at follow‐up were predictors of actual social distancing behaviour (Hagger et al., 2020). These findings are consistent with research into similar health‐focused behaviours, such as blood donation, where performance is likely to promote the health of others rather than the self (Conner et al., 2013). Descriptive norms are also highly influential in the adoption of social distancing behaviour: individuals adjust their own social distancing behaviour to be in line with their perceptions of how those around them are behaving (Norton et al., 2021). This effect can be attributed to the fact that the behaviour of others is perceived to be an informative cue to disease severity, which in turn is found to influence the extent to which individuals follow public health guidance (Norton et al., 2021).

### The importance of social identification

During times of crisis, individuals tend to unify and come together; social cohesion often arises following mass tragedies and natural disasters (e.g. Hawdon & Ryan, 2011). The perception of a shared and globally traumatizing threat such as COVID‐19—necessitating common and coordinated responses—enhances the perception that we are ‘all in the same boat’, regardless of previous divisions between social groups (Drury et al., 2016). This psychological assimilation of the personal and social self (Segal et al., 2018) is likely to enhance social identification (i.e. feelings of ‘oneness’) with the group. This more inclusive sense of social identity (e.g. national identity)—as discussed in the case of group norms—also facilitates the adoption of protective behaviours for the sake of the community, as opposed to merely for the self (Kramer & Brewer, 1984). Thus, social identification also serves as a key determinant of adherence to protective health behaviours. This extends to social distancing in the context of COVID‐19. Indeed, in the context of the UK lockdown—where individuals were ordered by government to ‘stay at home’ and permitted to leave for essential purposes only, such as for time‐limited daily exercise, obtaining food or medicine, providing care or working in an essential occupation (where working from home was not possible)—community‐level identification pre‐lockdown was found to directly predict adherence 2 months into the lockdown (Stevenson et al., 2021). Across 67 countries, individuals who identified more strongly with their nation reported consistently greater engagement in public health behaviours (e.g. social distancing and stricter hygiene) and support for public health policies (e.g. the closing of bars and restaurants) during the earlier months of the pandemic (van Bavel et al., 2022). These self‐reported relations were corroborated by actual behavioural data on physical mobility; higher national identification prior to the pandemic predicted lower movement during the early months (van Bavel et al., 2022). National‐level identification, as well as humanity‐level identification, was also found to predict enhanced well‐being during the 4th–7th weeks of lockdown, and family‐level identification predicted increased social distancing behaviour and perceptions of disease severity (Vignoles et al., 2021)—demonstrating that social identification is associated with protective actions.

It is likely that social identity not only predicts social distancing behaviour directly, but also interacts with other key social psychological variables—such as group norms. The social identity approach (Tajfel & Turner, 1979; Turner et al., 1987) proposes that individuals derive an important part of their self‐concept and self‐esteem from their group memberships, and therefore norms will be more likely to translate into behaviour when they are tied to an important group membership. Put differently, norm‐behaviour correspondence will be strengthened when the group membership defining the norm is a salient basis for self‐definition, and weakened when it is not (Terry et al., 1999; Terry & Hogg, 1996, 2001). The perceived group norms of a behaviourally relevant reference group, such as friends and university peers, predict intentions to engage in regular exercise, sun protection (Terry & Hogg, 1996), recycling (Terry et al., 1999), healthy eating (Åstrøm & Rise, 2001; Louis et al., 2007), sustainable agricultural practices (Fielding et al., 2008) and energy conservation (Costa & Kahn, 2013)—but only for those who identify strongly with the reference group.

Most research into the determinants of social distancing behaviour has adopted the TPB as a theoretical framework to investigate norm‐distancing relations, but very few empirical studies have acknowledged that social identity may moderate the strength of this particular relationship. One notable exception comes from Ryoo and Kim (2021), who investigated the effects of descriptive norms and social identity on social distancing behaviour. The research found that behavioural intentions conformed to the direction of the manipulated descriptive norm (i.e. to be either compliant or non‐compliant with social distancing regulations). Furthermore, individuals were found to defy social distancing guidelines when they perceived others to be non‐compliant—particularly when they identified strongly with the deviant others (i.e. those from the same university). These preliminary findings point to the fact that individuals will adjust their own social distancing behaviour in line with that of others when the ‘other’ represents an ingroup with which they strongly identify.

### Testing the social identity model of behavioural associations (SIMBA)

**FIGURE 1:**
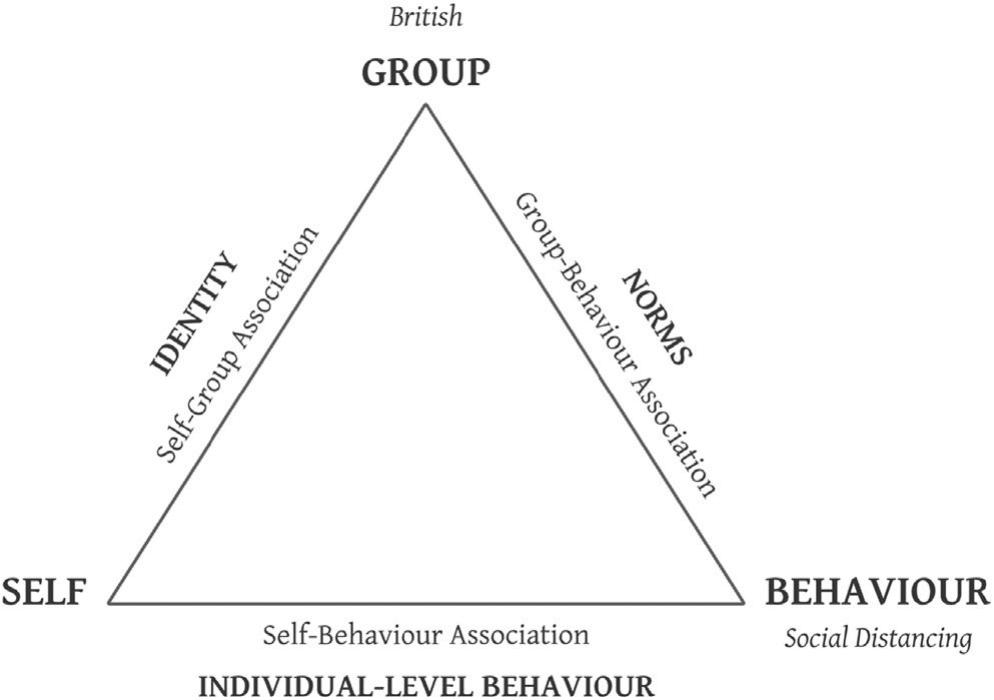
A representation of the Social Identity Model of Behavioural Associations (SIMBA). Each node/vertex of the triangle represents a psychological concept. The three associations measured in the design are identified along the triangle edges that join the vertices of the two relevant concepts. The self‐group association corresponds to a social identity, the group‐behaviour association corresponds to a group norm and the self‐behaviour association corresponds to individual‐level behaviour.

To date, most past research investigating the relationship between group norms and social distancing behaviour has measured this association in isolation, without appreciating that the social identity of the reference group is an important factor to consider, and has implications for the strength of the norm‐behaviour link. Individuals are likely to behave normatively only if they perceive that the norms are attached to a group with which they identify. Initial evidence for this interaction in the context of COVID‐19 social distancing behaviour, though scarce, can be found in the context of student social identity (Ryoo & Kim, 2021). However, research is yet to examine this relationship at more inclusive levels of social identity, such as national identity, which has been found to play an important role in motivating civic involvement (Huddy & Khatib, 2007) and engagement in costly behaviours that benefit other members of their national community (Kalin & Sambanis, 2018). In the context of the COVID‐19 pandemic, this level of identification is likely to have widespread implications for adherence to government guidelines at a population level—particularly given that salience of national identities may have increased during the pandemic as a result of travel bans, border closures and national task forces (Bieber, 2020). Moreover, while the norm‐distancing behaviour link has been established, research has called for further evidence regarding the long‐range predictors of social distancing behaviour (i.e. those that may predict social distancing over time), and the investigation of reciprocal effects via a more integrated model of behaviour (see Hagger et al., 2020). While recent evidence found subjective norms to predict changes in social distancing behaviour (e.g. avoiding crowds, quarantining, avoiding face‐to‐face contact, number of days in a week that people left their house) across 2 months at the beginning of the pandemic (Schumpe et al., 2022), reciprocal, interactive effects are yet to be explored. The current research tests the utility and validity of a Social Identity Model of Behavioural Associations (SIMBA, see Figure 1)—which proposes reciprocal, dynamic associations among British national identity, group norms and social distancing behaviour.

Integrating social identity theorizing (Tajfel, 1978; Tajfel & Turner, 1979; Turner et al., 1987) with the key methodological assumptions and theoretical underpinnings of Balanced Identity Theory (BIT; Greenwald et al., 2002), the SIMBA proposes a triadic relationship among social identity, group norms and individual‐level behaviour—conceptualizing them as cognitive *associations* 1 that can be measured both implicitly^2^ and explicitly (i.e. via self‐report). Research in the social identity tradition has often focused on explaining how social identity and group norms interact to predict individual behaviour (for a review see Hogg & Smith, 2007). However, the SIMBA conceptualizes the relationships among constructs of the social identity approach to be more dynamic than initially theorized. Social identity theory (Tajfel, 1978; Tajfel & Turner, 1979) acknowledges that behaviour may be predicted by the interaction of social identity and group norms, but it does not account for the possibility that social identity may be predicted by the interaction of group norms and behaviour. In other words, it usually focuses on behaviour as an outcome, but not a predictor of, other group‐level constructs. Equally, while self‐categorisation theory (Turner et al., 1987) proposes that social identification has a top‐down influence on the formation of group norms, it does not consider the bottom‐up process of identity formation through the validation of group norms (Postmes, Spears, et al., 2005) and group‐based behaviour (although see work regarding the Encapsulated Model of Social Identity in Collective Action [EMSICA]; Thomas et al., 2012, and the Identity‐Norm Nexus [INN]; Smith et al., 2015). The SIMBA addresses these gaps in the social identity approach—modelling the constructs as cognitive associations that are triadic, rather than bivariate—providing a more interactive and comprehensive explanation of group‐based behaviour.

The theoretical underpinnings of the SIMBA follow from the balance‐congruity principle—a principle drawing on Heider's (1946, 1958) balance theory and Osgood and Tannenbaum's (1955) congruity theory—appealing to the notion of a shared first order. That is, when two unlinked or weakly linked concepts share a first‐order link (i.e. when each of two nodes is linked to the same third node), the association between these two should strengthen (Greenwald et al., 2002). If, for example, the self‐group or the group‐behaviour link were to be of zero strength, then there would be no shared link, and no tendency to form the third (self‐behaviour) link. Rather than focusing solely upon identity and norms to predict behaviour, the three association measures can be interchanged in this multiplicative assumption—the strength of any one association is a product of the strength of the remaining two associations. The model proposes that while the combined strength of the identity‐norm association will predict behaviour, equally, the norm‐behaviour association will predict social identity, and the identity‐behaviour association will predict norms.

Integral to this proposition is the idea that individuals strive to maintain an element of cognitive consistency among concepts, and a failure to do so represents a state of imbalance‐dissonance. The notion of imbalance‐dissonance draws heavily on both balance theory (Heider, 1958) and cognitive dissonance theory (Festinger, 1957), and proposes that a state of psychological discomfort exists whenever an association between two variables fails to align with the strength and direction consistent with the relationship between each of those variables and a third variable. According to Heider (1958), inconsistency is typically resolved in the associative network by taking steps to change the polarity of relevant associative links—thereby resuming balance.

The balance‐congruity principle has received extensive support in the literature, particularly when measuring associations among the self‐group (identity), group‐attribute (attitudes, stereotyping) and self‐attribute (self‐esteem) constructs that form the focus of Greenwald et al.'s (2002) Balanced Identity Design (BID; for a review, see Cvencek et al., 2012; Cvencek et al., 2021). The BID was introduced alongside BIT (see Greenwald et al., 2002) as a triadic methodological framework for modelling the theory's predictions derived from the balance‐congruity principle—that the attitude towards a group (i.e. the group‐positive association) should develop in proportion to the product of the strengths of self‐positive (self‐esteem) and self‐group (identity) associations. The three association measures within the design can be exchanged in the same assumption, such that they demonstrate cognitive balance. One of the key strengths of BIT is that it possesses greater explanatory potential than traditional balance theory while retaining the core principles described previously. Whereas balance theory was designed to explain balance among *interpersonal relations* (i.e. it tells us about relations among individual people), BIT more broadly explains (and models, through the BID) *social knowledge*—which can be represented as ‘a network of variable‐strength associations among person concepts (including self and groups) and attributes (including valence)’ (Greenwald et al., 2002, p. 5).

However, by restricting its core focus to social psychology's affective (self‐esteem and attitude) and cognitive (stereotypes and self‐concept) constructs, it can be argued that BIT still captures only a very limited portion of our social knowledge network. That is, much like traditional balance theory, it tells us more about individual‐level processes than group‐level processes. Placing a heavier emphasis on modelling constructs not unrelated to, but primarily extending beyond, the self, recent research has demonstrated the utility of the SIMBA for the measurement of self‐group‐behaviour associations. In the context of drinking behaviour in relation to both student and British national identities, research has found support for the assumptions of the SIMBA to be consistently stronger on implicit, than explicit scale, measures (Hughes & Smith, 2024). However, this research demonstrated initial evidence that—when measured appropriately (i.e. using explicit measures with a zero‐point indicative of associative indifference, such as visual analogue scales)—the constructs of the SIMBA also demonstrate cognitive consistency at an explicit level (Hughes & Smith, 2024). Nevertheless, to date, the SIMBA has not been tested outside of the drinking context, nor is it clear whether new group‐level constructs quickly assume a balanced configuration, or require a gradual process of dissonance reduction—thereby necessitating validation in a novel behavioural context.

### The present research

The present research primarily tests the utility and validity of the SIMBA for the measurement of interrelations among British national identity, group norms and social distancing behaviour, both implicitly and explicitly (i.e. via self‐report). While previous research has provided initial correlational support for the assumptions of the model (e.g. balance‐congruity among self‐group‐behaviour concepts; Hughes & Smith, 2024), as previously noted, all previous tests have been conducted in the context of drinking behaviour in relation to British national and student identities. Therefore, tests of the model outside of this behavioural context are essential in establishing its broader generalisability and applicability at this stage of development.

Moreover, the pandemic created a unique behavioural context to test the assumptions of the SIMBA. All previous correlational tests of the model for the measurement of associations have been conducted in the context of established behaviours that demonstrate strong, pre‐existing associations with particular group memberships (see Hughes & Smith, 2024). In contrast, social distancing represents a behaviour that is novel in terms of the terminology used to describe the behaviour, the prevalence of its performance, and its lack of group affiliation pre‐pandemic. Consequently, the present research also goes beyond, and therefore extends, previous tests of the model by shedding light on the value and utility of the SIMBA for measuring new associations as they emerge. In demonstrating support for the balance‐congruity principle (Greenwald et al., 2002) both implicitly and explicitly, the research bolsters the supposition that, while the constructs of social identity and group norms are typically measured via Likert scales, explicit confirmation of balance‐congruity among self‐group‐behaviour triads is strongest on self‐report measures possessing a rational zero‐point. This is as typically found within the balanced identity literature (see Cvencek et al., 2021) and found in previous correlational tests of the SIMBA (Hughes & Smith, 2024). Identifying that newly emergent associations also arrange themselves in a mutually interactive manner at both levels of measurement provides an important foundation and rationale for future experimental tests of the model. Tests of this kind will be essential in establishing the SIMBA as a model that both describes *and* predicts the measurement and formation of group‐based behaviour.

The changing behavioural landscape of the pandemic, characterized by changes in governmental guidance and behavioural restrictions, also created a unique opportunity to explore the extent to which associations among constructs hold longitudinally, and maintain their configurations of balance‐congruity; tests of the SIMBA, and of balanced identity more broadly, have not determined this. Implicit measures in particular have been found to show particularly low levels of temporal stability when changes in the broader context activate different associations at different measurement points (see Gschwendner et al., 2008; Rydell & Gawronski, 2009). Therefore, it can be expected that associative strength will fluctuate to the extent that the associations are, or are not, reinforced in a consistent manner throughout the pandemic. The present research extends previous single timepoint tests of the SIMBA (see Hughes & Smith, 2024) by exploring self‐group‐behaviour associations cross‐sectionally at two different points throughout the pandemic—the beginning of May 2020 and the end of June 2020—while also examining whether interactions among any two of the constructs measured earlier in the pandemic predict the third construct months later. The research thereby investigates the extent to which British national identity, group norms and social distancing behaviour demonstrate reciprocal relationships over time and serve as long‐range predictors—as previous research has called for (see Hagger et al., 2020). In doing so, it also offers practical implications. Identification of these potentially modifiable long‐term determinants of social distancing behaviour may provide insights to inform interventions that promote adherence to public health guidelines. In accordance with the balance‐congruity principle, we tested the following hypotheses:^3^

**H1**: Individuals will demonstrate consistent positive associations among self‐group, group‐behaviour, and self‐behaviour constructs at (a) Time 1 and (b) Time 2.

**H2** 4: Associative strength will change between Time 1 and Time 2, both (a) implicitly and (b) explicitly.

**H3**: Time 2 post‐lockdown social distancing behaviour will be predicted positively by Time 1 post‐lockdown behavioural intentions on explicit measures.

**H4**: Individual behaviour will be predicted by the interaction between social identity and group norms at (a) Time 1 and (b) Time 2 both (i) implicitly and (ii) explicitly.

**H5**: Group norms will be predicted by the interaction between individual behaviour and social identity at (a) Time 1 and (b) Time 2 both (i) implicitly and (ii) explicitly.

**H6**: Social identity will be predicted by the interaction between individual behaviour and group norms at (a) Time 1 and (b) Time 2 both (i) implicitly and (ii) explicitly.

**H7** 5: Time 2 (a) individual behaviour, (b) group norms, and (c) social identity will be predicted by the interaction between the change scores of the remaining two variables both (i) implicitly and (ii) explicitly.

## METHOD

### Participants and design

The two‐part longitudinal study recruited 151^6^ participants (52 males, 98 females, 1 missing; *M*_*age*_ = 39.94, *SD* = 13.90) via Prolific. Participants were pre‐screened to ensure that they were of self‐identified British (UK) nationality. The Time 1 study commenced on 9 May 2020, when the UK was in full lockdown—meaning that individuals were only permitted to leave home to shop for basic necessities, travel to work when home working was not possible or to exercise once a day. Of those entering the study at Time 1, 136^7^ participants (47 males, 88 females, 1 missing; *M*_*age*_ = 40.41, *SD* = 13.89) completed Time 2; this study commenced on 21 June 2020 once the full lockdown had lifted. For their participation, individuals received remuneration of £2.50 per timepoint completed, plus an additional 50p bonus payment for completing both timepoints. Remuneration was awarded separately for each timepoint once full completion was confirmed, providing that Prolific ID was entered correctly and consistently throughout the study; no participants were excluded on this basis.

### Implicit association test measures

Participants completed three word‐and‐picture Implicit Association Tests (IATs; Greenwald et al., 1998) in the following order^8^: one measuring the group‐behaviour association (contrasting British and non‐British images, as well as socially distanced and non‐socially distanced images), another measuring the self‐behaviour association (contrasting self and other words, as well as socially distanced and non‐socially distanced images), and another measuring the self‐group association (contrasting self and other words, as well as British and non‐British images). The content of pictorial IAT stimuli was matched across each opposing category title (i.e. British/non‐British) and, aside from ‘British’ stimuli, aimed to be geographically ambiguous.

Each of the three IATs trialled these word‐and‐picture associations across seven test blocks. Participants had to categorize stimuli associated with the left‐hand category by pressing the ‘E’ key, and stimuli associated with the right‐hand category by pressing the ‘I’ key. Practice blocks one, two and five were single‐category ‘practice’ blocks, where participants sorted target or attribute stimuli into different category titles. The remaining blocks were ‘critical’ combined blocks, where target and attribute category titles appeared together in both compatible and incompatible configurations.

The initial combined block was randomized between participants—first compatible for half of participants, and first incompatible for half of participants—providing four possible starting configurations. Each participant was allocated randomly to one of these four permutations for each IAT. The two compatible configurations included Target A on the right (e.g. social distancing) with attribute A (e.g. British), and Target A on the left with attribute A. The two incompatible configurations included Target A (e.g. social distancing) on the right with attribute B (e.g. non‐British), and Target A on the left with attribute B. The underlying principle of the IAT is that it is easier to give the same response to items representing categories that are associated in memory than to ones representing categories that are not associated. Therefore, the extent to which an individual is faster in either compatible or incompatible combined blocks is an indication of the strength of their implicit association.

### Explicit measures^9^

#### Demographics

At the end of the Time 1 survey, the demographics of age, gender identity, key worker status (i.e. those whose work was critical to the COVID‐19 response; a list of all occupations meeting key worker status was provided by the Cabinet Office, 2020) and COVID‐19 risk status were collected. In addition to these items, Time 2 also collected demographic information regarding political orientation, compliance with government guidelines and region of residency throughout lockdown. At both timepoints, participants were asked whether they, or anyone else in their household, had contracted COVID‐19.

#### Social distancing behaviour

Social distancing behaviour—steps that one can take to reduce social interaction—was measured using nine items regarding the use of transport, working arrangements, socializing and communications. Participants were required to rate how often they engaged in social distancing behaviours such as ‘Staying 2 m (6ft) apart from anyone outside of your own household at all times when leaving the house’ and ‘Avoiding gatherings with friends living outside of your own household’ on a 5‐point Likert scale, with 1 being ‘never’ and 5 being ‘always’. At Time 1 only, participants were also required to rate how often they intended to engage in the same nine social distancing behaviours once the full UK lockdown was lifted.

Following an oblique rotation confirming that factors were not correlated, a principal component analysis (PCA) using orthogonal varimax rotation was performed on these nine items at each timepoint. Items 1 (‘Avoiding non‐essential use of public transport’) and 2 (‘Working from home when possible’) were dropped from the analysis, as they did not fit with any of the other items on any suggested factor. Following the examination of a scree plot, the analysis proposed a two‐factor solution—revealing that two aspects of social distancing behaviour were empirically distinct. The two factors accounted for 55% of the variance at Time 1, and 59% of the variance at Time 2. The five items assessing social avoidance behaviours loaded onto the first factor (T1 eigenvalue = 2.52, factor loadings = .56–.78, T2 eigenvalue = 2.83, factor loadings = .62–.81). The two items assessing use of online technology loaded onto the second factor (T1 eigenvalue = 1.34, factor loadings = .75–.85, T2 eigenvalue = 1.28, factor loadings .78–.85). Thus, the seven items formed two scales of social avoidance behaviour (T1 *α =* .70, T2 *α =* .78) and use of online technology. Scales were derived by taking a mean of responses; the social avoidance component of social distancing behaviour was the focus of this research.

In addition, participants were required to complete one continuous 101‐point visual analogue scale (VAS). Participants were asked to drag a slider indicating how they typically think of their behaviour in the context of COVID‐19, with −50 being ‘not engaging in social distancing’, +50 being ‘engaging in social distancing’ and 0 indicating a lack of inclination to engage in either behaviour.

#### Social identification

Identification with the British national identity was measured using four items adapted from Doosje et al. (1995). Participants were required to rate items such as ‘I feel strong ties with other British people’ on a 7‐point Likert scale, with 1 being ‘strongly disagree’ and 7 being ‘strongly agree’. A mean of the responses to the four items above was taken to create a uni‐dimensional measure of identification (T1 and T2 *α =* .94), with higher scores representing greater identification with the British national identity.

In addition, participants were required to complete one continuous 101‐point VAS. Participants were asked to drag a slider indicating how they generally view themselves, with −50 being ‘non‐British’, +50 being ‘British’ and 0 indicating absence of preference.

#### Group norms

The descriptive norms associated with the British identity were measured using five items adapted from Tarrant and Smith (2011). Participants were asked to rate items such as ‘Social distancing is something that British people typically do’ on a 7‐point Likert scale, with 1 being ‘strongly disagree’ and 7 being ‘strongly agree’. A mean of the responses to the five items above was taken to create a uni‐dimensional measure of norms (T1 *α =* .86, T2 *α =* .92), with higher scores representing a greater perceived relation between British individuals and social distancing behaviour.

In addition, participants were required to complete one continuous 101‐point VAS. Participants were asked to drag a slider to indicating how the British are typically seen as behaving in the context of COVID‐19, with −50 being ‘not engaging in social distancing’, +50 being ‘engaging in social distancing’ and 0 indicating absence of preference.

### Procedure

Following ethical approval, the study was advertised on Prolific. When entering the Time 1 study during the full UK lockdown, participants were informed that the research aimed to examine different types of group membership and health behaviours in the context of COVID‐19. After indicating their informed consent, participants completed the three group‐behaviour (group norm), self‐behaviour (individual behaviour) and self‐group (social identity) IATs in this order. For the purpose of matching responses from the same participant across IATs, participants were required to enter their unique Prolific ID before each task. They then went on to complete explicit self‐report measures of social distancing behaviour, post‐lockdown behavioural intentions, social identification, group norms and well‐being in this order. Demographic information was collected, and participants were thanked for participating in the first part of the research.

Providing that a Prolific ID had been entered correctly and consistently before each task at Time 1, participants were invited to complete the Time 2 study 6 weeks later—once the full UK lockdown had lifted, but the government message was that individuals should continue to socially distance from others where possible. Here, the Time 1 study design was replicated, excluding the measure of behavioural intentions. Finally, participants were debriefed and provided with the contact details of the researchers and the chair of ethics to address any queries. Participants were also provided with national and international COVID‐19 resources for further information regarding local guidelines and health advice.

### Transparency and openness

Study hypotheses, materials and data analysis strategy were pre‐registered. Materials, analysis code and data are also available on the Open Science Framework (OSF). We report how we determined our sample size, our measures, our data analysis strategy and all data exclusions.

## RESULTS

### Data analysis strategy^10^

Descriptive statistics are presented for participant demographics. For all implicit and explicit measures, correlational analyses were performed to establish whether a relationship existed between the measures (i.e. whether they produced similar outcomes).

One‐sample *t*‐tests (relating to H1) were conducted on the mean scores from the IAT (*D‐*scores generated by the IATGEN analysis tool; Carpenter et al., 2019) and VAS measures of each association to determine whether they were significantly different from zero. A positive *D‐*score indicates an association between compatible target and attribute categories, such as ‘social distancing’ and ‘British’. For all implicit and explicit measures, paired‐samples *t*‐tests were conducted to reveal any changes in associative strength between timepoints (i.e. H2). To examine whether post‐lockdown behavioural intentions at Time 1 predict self‐reported post‐lockdown behaviour at Time 2 (i.e. H3), a linear regression was performed.

**FIGURE 2:**
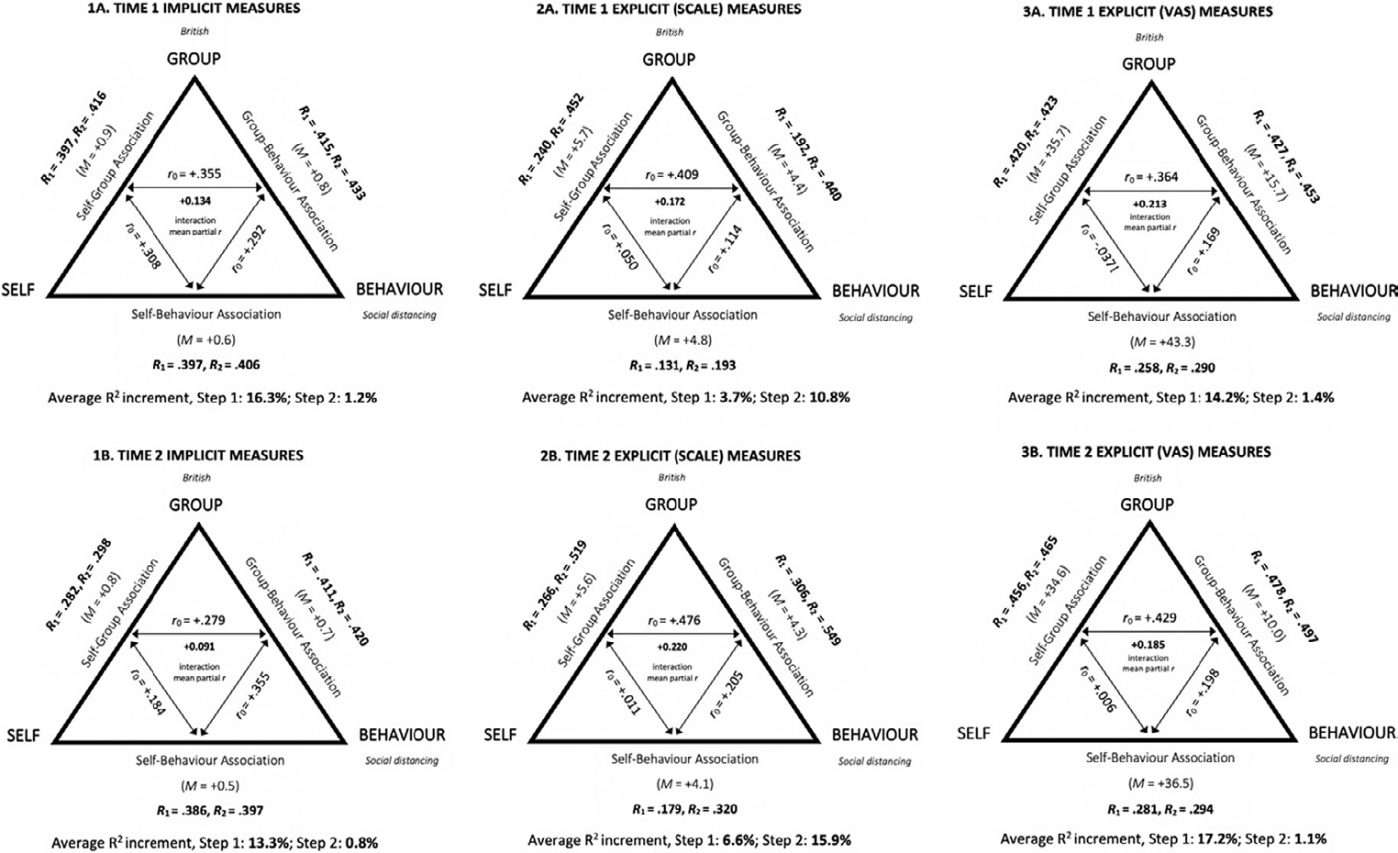
Summary of statistical tests for all implicit and explicit measures. In the case of implicit measures (1A and 1B), *M* is representative of *D‐*score mean. Each zero‐order correlation (*r*_0_) on an edge of an inner triangle relates the two association measures pointed to by the adjoining edge's two arrows. *R*_1_ and *R*_2_ identify the multiple regression correlation coefficients produced, respectively, in the first and second steps of the regression analyses. Exclamation marks (!) follow *r*_0_ values that are opposite in sign from prediction and *R*_1_ values that are associated with opposite‐from‐predicted (i.e. negative) interaction effect coefficients.

To test the balance‐congruity principle for all implicit and explicit measures (i.e. H4 through H6), a series of two‐step moderated regressions were conducted with reverse entering of the direct and interaction effects (see Greenwald et al., 2002). Centred means were used for the explicit scale measures. Implicit and explicit (VAS) measures were not centred for moderated regressions under the assumption that they have a rational zero‐point (for a detailed argument, see Greenwald et al., 2006). The validity of this method for testing balance‐congruity has been described by Greenwald et al. (2002), with some elaboration in Greenwald et al. (2006). Zero‐order correlations (*r*_0_) aid in descriptively supporting the extent to which the strength of any one association can be predicted by the combined strength of the remaining two. Zero‐order correlations between two association measures are consistent with the balance‐congruity principle where they possess the same sign as the mean value of the remaining association measure. Therefore, when any variable in SIMBA significantly differs from zero and is polarized towards its high end, the zero‐order correlation between the other two variables should be positive (see Greenwald et al., 2002). A summary of results pertaining to these predictions can be found in Figure 2.

To provide a quantitative indicator of the magnitude of confirmation with regards to the balance‐congruity principle, data resulting from the moderated regressions were then further analysed using a within‐study meta‐analysis^11^ (see Cvencek et al., 2021). The within‐study meta‐analysis combined the three correlation effect sizes produced by Test 1, and (separately) those produced by Test 2 of the moderated regressions. The Test 1 effect size measure is the coefficient of the product term entered at Step 1, converted to an *r* value. The Test 2 effect size measure is the coefficient of the product term at Step 2, converted to a signed partial correlation (*pr*). For each of the three types of criterion measure (self‐group, group‐behaviour and self‐behaviour), and separately for self‐report and IAT measures, the three effect sizes were transformed to Fisher *Z* values and aggregated in a random‐effects, within‐study meta‐analysis. The within‐study meta‐analysis uses only data from Tests 1 and 2 of the moderated regression because (a) the *r* associated with Test 1 can be interpreted as a basic test of fit of a multiplicative theoretical model, and (b) the *pr* associated with Test 2 can be interpreted as an index of fit of a pure multiplicative theoretical model. To demonstrate support for a pure multiplicative theoretical model, entry of the multiplicative product on Step 1 of the moderated regression should account for significant criterion variance, and no significant portion should be accounted for when the two predictors are entered individually on Step 2 (see Cvencek et al., 2021). Cochran's *Q* test for heterogeneity in meta‐analysis was conducted to examine the degree of variation across outcome variables—the null hypothesis being that all outcome measures estimate the same multiplicative effect, and are therefore homogenous.

For longitudinal analyses (i.e. H7), a change score (Time 2–Time 1) was calculated for each of the implicit and explicit associations. Multiple linear regressions were then conducted. The Time 2 outcome variable for each association at each measurement level was predicted by the remaining two change score variables, and the interaction between them, while also controlling for the outcome variable at Time 1.^12^

### Participant characteristics

Regarding political orientation, the sample was centre‐left leaning; categorical demographic information detailing other participant characteristics is presented in Table 1. From this information, it is evident that the majority of the sample at both timepoints had not personally contracted COVID‐19, nor known of anyone in their household to have contracted the virus. Around a third of the sample at both timepoints were of key worker status, and the majority were not classified as being high risk. Regarding region of residence over the course of lockdown, the sample was geographically well distributed across the UK—the largest number coming from the South East (16%). Overall, the majority of participants reported complying with government guidelines to the same extent post‐lockdown as during lockdown; 22% reported being less compliant, with only 7% reporting increased compliance.

**TABLE 1:** Detailed demographic information.

		Time 1 (*N* = 151)			Time 2 (*N* = 136)	
		*N*	*%*		*N*	*%*
Had COVID‐19	Yes, tested positive	1	0.7		1	0.7
	Yes, symptoms	18	11.9		12	8.9
	No	132	87.4		122	90.4
Someone in house had COVID‐19	Yes, tested positive	1	0.7		1	0.7
	Yes, symptoms	11	7.3		11	8.1
	No	139	92.1		123	91.1
Classified as a key worker	Yes	51	33.8		44	32.4
	No	100	66.2		92	67.6
Classified as high risk	Yes	17	11.3		14	10.3
	No	134	88.7		122	89.7
Change in compliance	More compliant	–	–		10	7.4
	Less compliant	–	–		30	22.1
	Equally compliant	–	–		96	70.6
Region during lockdown	Greater London	–	–		9	6.6
	South East	–	–		22	16.2
	South West	–	–		17	12.5
	West Midlands	–	–		12	8.8
	North West	–	–		14	10.3
	North East	–	–		6	4.4
	Yorkshire and the Humber	–	–		19	14
	East Midlands	–	–		19	14
	East of England	–	–		14	10.3
	Other	–	–		4	2.0

### Correlations among measures

Bivariate correlations among all implicit and explicit measures (see Table 2) revealed that when examining within‐construct relations between timepoints, all corresponding Time 1 and Time 2 measures of social identity, group norms and behaviour were positively and significantly correlated—with the exception of the implicit measure of behaviour, which, though positive in direction, did not reach conventional levels of significance. Equally, when examining within‐construct relations between measurement levels, the corresponding implicit and explicit measures were positively correlated—with the exception of Time 2 implicit behaviour, which was negatively correlated with Time 1 explicit (VAS) behaviour, and significantly so with all Time 2 explicit measures of behaviour. All corresponding explicit scale and VAS measures were positively correlated and highly significant.

**TABLE 2:** Bivariate correlations among all implicit and explicit measures.

Variable	1	2	3	4	5	6	7	8	9	10	11	12	13	14	15	16	17	18
1. Implicit norms (T1)	—																	
2. Implicit norms (T2)	.39**	—																
3. VAS norms (T1)	.05	.13	—															
4. VAS norms (T2)	.09	.16	.59**	—														
5. Explicit norms (T1)	.11	.18*	.62**	.48**	—													
6. Explicit norms (T2)	.06	.07	.55**	.75**	.56**	—												
7. Implicit behav (T1)	.30**	.23**	.07	.13	.07	.05	—											
8. Implicit behav (T2)	.17	.36**	−.02	.004	.05	−.03	.15	—										
9. VAS behav (T1)	.03	.05	.17*	.03	.05	.06	.10	−.04	—									
10. VAS behav (T2)	−.02	.02	.06	.20*	.09	.13	.06	−.19*	.46**	—								
11. Explicit behav (T1)	.08	.21*	.02	.11	.11	.13	.10	.04	.45**	.48**	—							
12. Explicit behav (T2)	.09	−.02	.05	.17*	.12	.21*	.17	−.19*	.25**	.67**	.45**	—						
13. Implicit ident (T1)	.35**	.32**	.06	.11	−.03	.06	.31**	.20*	.03	.02	.05	.02	—					
14. Implicit ident (T2)	.30**	.28**	.14	.21*	.19*	.15	.26**	.18*	−.001	−.02	−.01	−.03	.51**	—				
15. VAS ident (T1)	.23**	.32**	.36**	.41**	.36**	.41**	.15	.02	−.04	−.04	.00	.03	.26**	.37**	—			
16. VAS ident (T2)	.22**	.24**	.42**	.43**	.27**	.35**	.13	.02	−.01	.01	.05	−.01	.23**	.30**	.75**	—		
17. Explicit ident (T1)	.24**	.28**	.30**	.43**	.41**	.47**	.14	.08	−.07	−.08	.05	−.03	.19*	.30**	.76**	.62**	—	
18. Explicit ident (T2)	.19*	.21*	.45**	.50**	.38**	.48**	.18*	.05	−.02	−.02	.07	.01	.20*	.40**	.69**	.85**	.73**	—

*Notes*: The term explicit here is used to refer to Likert scales of a particular construct, though VAS are also explicit measures.

*p < .05. **p < .01. (Two‐tailed test).

When examining between‐construct relations within measurement levels, all implicit measures were positively and—with the exception of the association between Time 1 implicit norms and Time 2 implicit behaviour—significantly correlated. Similarly, the majority of explicit measures were positively correlated. However, there were some very slight negative correlations, though none significant; explicit (VAS) behaviour demonstrated a negative correlation with all explicit measures of identity at both timepoints, and Time 2 explicit (VAS) behaviour demonstrated a negative correlation with Time 2 explicit (VAS) identity and Time 1 explicit (scale) identity.

In sum, measures of social identity, group norms and behaviour tend to be positively correlated with one another across time (i.e. when measured at Time 1 and Time 2) and across measurement levels (i.e. when measured implicitly and explicitly). Implicit measures of the different constructs also tend to be positively correlated with one another, as do explicit measures.

All implicit and explicit measures were polarized towards high values, and as expected from this finding, the majority of zero‐order correlations were positive (see Figure 2).

### One‐sample *t*‐tests

#### Implicit measures

One‐sample *t*‐tests establishing the presence of implicit associations among constructs revealed that the mean Time 1 *D‐*scores for social identity *t*(147) = 29.14, *p* < .001, *d* = 2.40, group norms *t*(150) = 30.34, *p* < .001, *d* = 2.47 and behaviour *t*(148) = 19.35, *p* < .001, *d* = 1.59 were all significantly different from zero. Therefore, in adherence with standardized cut‐off values for association strength^13^ (Cohen, 1977), participants demonstrated ‘strong’ positive associations among self‐group (*D* = .88, *SD = *.37) and group‐behaviour (*D =* .80, *SD* = .33) constructs, with ‘moderate’ positive associations demonstrated among self‐behaviour constructs (*D* = .59, *SD =* .37).

Similarly, mean Time 2 *D‐*scores for social identity *t*(130) = 27.74, *p* < .001, *d* = 2.42, group norms *t*(133) = 20.36, *p* < .001, *d* = 1.76 and behaviour *t*(132) = 18.45, *p* < .001, *d* = 1.60 were all significantly different from zero. The corresponding Time 1 and Time 2 associations were also of equivalent associative strength; participants demonstrated ‘strong’ positive associations among self‐group (*D* = .84, *SD* = .35) and group‐behaviour (*D* = .68, *SD* = .39) constructs, with ‘moderate’ positive associations demonstrated among self‐behaviour constructs (*D =* .53, *SD* = .33).

#### Explicit (VAS) measures

One‐sample *t*‐tests establishing the presence of explicit associations among constructs revealed that the mean Time 1 VAS scores for social identity *t*(150) = 22.39, *p* < .001, *d* = 1.82, group norms *t*(150) = 7.56, *p* < .001, *d* = .62 and behaviour *t*(149) = 35.78, *p* < .001, *d* = 2.92 were all positive and significantly different from zero. Similarly, mean Time 2 VAS scores for social identity *t*(134) = 19.23, *p* < .001, *d* = 1.66, group norms *t*(135) = 4.54, *p* < .001, *d* = .39 and behaviour *t*(135) = 24.71, *p* < .001, *d* = 2.12 were all positive and significantly different from zero. Participants saw themselves as being British (Time 1: *M* = 35.66, *SD* = 19.57; Time 2: *M* = 34.56, *SD* = 20.88), thought of the British in terms of engaging in social distancing behaviour (Time 1: *M* = 15.74, *SD* = 25.56; Time 2: *M* = 9.97, *SD* = 25.62) and also thought of themselves in terms of engaging in social distancing behaviour (Time 1: *M* = 43.31, *SD* = 14.83; Time 2: *M* = 36.46, *SD* = 17.21).

### Paired‐sample *t*‐tests

Paired‐sample *t*‐tests investigating change in associative strength between timepoints (see Table 3) revealed that there was a significant decrease in the strength of the group‐behaviour association at both the implicit and explicit (VAS) levels. Regarding the self‐behaviour associations, there also was a significant decrease in associative strength at the explicit level only on both VAS and Likert measures. However, there was no change in the strength of the self‐group association at either the implicit or explicit level.

**TABLE 3:** Change in associative strength between Time 1 and Time 2 for all measures.

	Association	*M*_*diff*_	*SD*	*t*	*Df*	*p*	Cohen's *d*
Implicit	Norms	.**14**	.**40**	**4.11**	**133**	**<.001**	.**36**
	Behaviour	.06	.47	1.48	132	.141	.13
	Identity	.03	.34	1.15	128	.253	.10
Explicit (VAS)	Norms	**6.88**	**22.98**	**3.49**	**135**	.**001**	.**30**
	Behaviour	**7.46**	**16.08**	**5.39**	**134**	**<.001**	.**46**
	Identity	.83	14.48	.67	134	.507	.06
Explicit (Scale)	Norms	.15	1.14	1.55	135	.123	.13
	Behaviour	.**68**	.**65**	**12.31**	**135**	**<.001**	**1.06**
	Identity	.12	.96	1.50	135	.136	.13

*Note*: Cells in bold represent significant changes in associative strength.

### Predicting post‐lockdown behaviour

A linear regression to predict post‐lockdown behaviour from behavioural intentions during lockdown revealed that Time 1 behavioural intentions significantly predicted Time 2 social distancing behaviour *b* = .34, *t*(133) = 5.63, *p <* .001; participants' post‐lockdown behaviour increased .34 for each one‐point increase in their ratings of post‐lockdown behavioural intentions. Behavioural intentions also explained a significant proportion of the variance in Time 2 self‐reported social distancing behaviour *R* ^2^ = .19, *F*(1,133) = 31.67, *p* < .001.

### Testing the SIMBA: Within‐study meta‐analyses

#### Time 1

For all implicit and explicit measures, the weighted aggregate effect sizes (see Table 4) were significant for both Test 1 and Test 2. Therefore, data provide confirmation for SIMBA's predictions of fitting a pure multiplicative model—whereby data can be fit entirely by the interaction term. However, the effect sizes obtained from VAS were larger than those obtained from Likert scale measures. Heterogeneity was non‐significant for both Test 1 and Test 2, suggesting no variation across the outcome measures of social identity, group norms or individual‐level behaviour.^14^

**TABLE 4:** Average within‐study effect sizes from Test 1 and Test 2—Time 1.

*N*		Test 1			Test 2		
		*r*	MOE	*Q*	*r*	MOE	*Q*
148	Implicit	.403***	.**079**	.045	.135**	.**095**	2.133
151	Explicit (VAS)	.371***	.**106**	3.492	.213***	.**089**	.010
151	Explicit (Scale)	.188***	.**089**	.949	.172***	.**090**	.355

*Note*: Cells in bold under the columns reporting *r* and MOE represent results consistent with predictions of the SIMBA.

Alpha levels for *r* and *Q* are **p ≤ .01, ***p ≤ .001.

#### Time 2

For implicit measures, the weighted aggregate effect sizes (see Table 5) were significant for Test 1 but were only of marginal significance for Test 2 (*p* = .072). Therefore, data provide confirmation for SIMBA's predictions of fitting a multiplicative model—though this is not a *pure* multiplicative fit, due to the marginal result of Test 2. In other words, data are not fit entirely by the interaction term.

**TABLE 5:** Average within‐study effect sizes from Test 1 and Test 2—Time 2.

*N*		Test 1			Test 2		
		*r*	MOE	*Q*	*r*	MOE	*Q*
131	Implicit	.361***	.**087**	1.546	.092	.099	1.041
135	Explicit (VAS)	.408***	.**119**	4.217	.185***	.**095**	.067
136	Explicit (Scale)	.251***	.**092**	1.266	.220***	.**093**	.030

*Note*: Cells in bold under the columns reporting *r* and MOE represent results consistent with predictions of the SIMBA.

Alpha levels for *r* and *Q* are ***p ≤ .001.

For both explicit measures, the weighted aggregate effect sizes were significant for Test 1 and Test 2, although the Test 1 effect sizes obtained from VAS were larger than those obtained from Likert scale measures—as was also evident in the Time 1 data. Therefore, data provide confirmation for SIMBA's predictions of fitting a pure multiplicative model—whereby data can be fit entirely by the interaction term.

Across all measurement levels, heterogeneity was non‐significant for both Test 1 and Test 2, suggesting no variation across the outcome measures.

### Longitudinal analyses

A multiple linear regression was conducted for each Time 2 outcome variable (i.e. implicit and explicit behaviour, identity and group norms) to investigate whether they are predicted by the interaction between the change scores of the remaining two variables—while also controlling for the direct effects of the two change score variables, and the outcome variable at Time 1. Table 6 provides a summary of these longitudinal analyses for all implicit and explicit data.

**TABLE 6:** Summary of longitudinal multiple linear regression analyses for all implicit and explicit measures.

	Criterion (Time 2)	*R* ^2^	*F*	Direct effect 1	Direct effect 2	Direct effect 3	Interaction
Implicit	Behaviour	.07	2.37	.14	.01	.17*	−.15
	Identity	.26	11.05***	.46***	−.003	−.03	−.11
	Norms	.15	5.32***	.40***	−.14	.09	−.24
Explicit (VAS)	Behaviour	.26	11.18***	.65***	.09	.16**	.002
	Identity	.57	42.32***	.78***	−.02	.05	.00
	Norms	.38	20.09***	.61***	−.06	.33**	−.01
Explicit (Scale)	Behaviour	.21	8.94***	.90***	.03	.06	.03
	Identity	.54	38.66***	.68***	.19*	.06	.15
	Norms	.33	16.07***	.60***	.08	.24	.06

*Note*: Alpha levels are *p < .05, **p < .01, ***p < .001.

Regression coefficients were reported as unstandardized beta.

Direct effect 1 = criterion variable measured at Time 1.

Direct effect 2 = identity change score and Direct effect 3 = norms change score where criterion is behaviour.

Direct effect 2 = norms change score and Direct effect 3 = behaviour change score where the criterion is identity.

Direct effect 2 = identity change score and Direct effect 3 = behaviour change score where the criterion is norms.

Interaction term = Direct effect 2*Direct effect 3.

With the exception of the prediction of implicit behaviour, the Time 2 criterion variables were predicted by their respective regression equations; each model was highly significant (*ps* < .001). For each of these regression equations, the outcome variable at Time 1 consistently and significantly contributed to the model; the overall significance of each model could overwhelmingly be attributed to this. Regarding the implicit measures, the Time 1 outcome variable was the only direct effect significantly contributing to the two significant models. Regarding the explicit measures, the group norms change score significantly predicted explicit (VAS) behaviour at Time 2; participants' explicit (VAS) behaviour at Time 2 increased .16 for each one‐point increase in their group norms change score. Equally, the behaviour change score significantly predicted explicit (VAS) group norms at Time 2; participants' explicit (VAS) group norms at Time 2 increased .33 for each one‐point increase in their behaviour change score. The group norms change score also significantly predicted explicit identity (scale) at Time 2; participants' explicit (scale) identity at Time 2 increased .19 for each one‐point increase in their group norms change score. However, contrary to H7, the change score interaction terms did not significantly contribute to any of the models; each outcome variable at Time 2 was not significantly predicted by the interaction between the change scores of the two remaining variables.

## DISCUSSION

The present research tested the theoretical framework of the SIMBA (Hughes & Smith, 2024) for the measurement of reciprocal implicit and explicit associations among British national identity, group norms and social distancing behaviour—both cross sectionally and over time—in the context of the COVID‐19 pandemic. The research also explored changes in the strength of these associations between two points during the pandemic. At both timepoints, participants demonstrated positive associations among self‐group, group‐behaviour and self‐behaviour concepts (H1). Evidence regarding the change in associative strength was mixed. The self‐group association remained stable at both an implicit and explicit level, but the self‐behaviour association decreased significantly on explicit self‐report measures only, and the group‐behaviour association significantly decreased both implicitly and explicitly. Therefore, H2 was only partially supported. Nevertheless, post‐lockdown behavioural intentions during the full UK lockdown were found to significantly predict self‐reported social distancing behaviour later in the pandemic, when strict guidelines had been renounced (H3). With regards to balance‐congruity, support for the principle was strong at both timepoints—the strength of any one association was predicted by the combined strength of the remaining two, both implicitly and explicitly (H4 through H6). This interpretation is further supported by the test for heterogeneity in meta‐analysis, which was consistently significant across timepoints—suggesting there to be no variation across the outcome measures of social identity, group norms and individual‐level behaviour. However, the strength of any one association, as measured post‐lockdown, was not predicted by the interaction between the change scores of the remaining two, meaning that H7 was not supported.

Our results demonstrate support for the balance‐congruity principle on both implicit and explicit measures. Nevertheless, the effect sizes from the explicit VAS measures—particularly those from Test 1 of the within‐study meta‐analyses—were substantially larger than from the Likert measures. Therefore, our findings are in line with our previous tests of the SIMBA—where explicit associations were measured using explicit self‐report measures with a rational and theoretically meaningful zero‐point (i.e. those where zero is indicative of associative indifference among concepts, as is the case for VAS, see Hughes & Smith, 2024). In providing a comparison between explicit Likert and VAS, our findings bolster the supposition that while the constructs of social identity and group norms are typically measured via Likert scales, explicit confirmation of balance‐congruity among self‐group‐behaviour triads is strongest on measures possessing a rational zero‐point—as is typically found within the balanced identity literature (see Cvencek et al., 2021).

The confirmation of balance on explicit self‐report measures of social identity, group norms and individual‐level behaviour is also in line with traditional social identity theorizing (Tajfel, 1978; Tajfel & Turner, 1979). Our findings replicate the traditional social identity by group norm interaction in predicting behaviour, which has been demonstrated in numerous health‐related contexts (e.g. Louis et al., 2007; Terry & Hogg, 1996)—including COVID‐19 social distancing behaviour (Ryoo & Kim, 2021)—but also demonstrate that all social identity variables can be exchanged in the same multiplicative assumption. Importantly, in the context of the pandemic, the relationship between distancing norms and social distancing behaviour—as moderated by social identity—is not uni‐directional. Rather, it is reciprocal, with the perception of distancing norms predicting national identity and vice versa—as moderated by an individual's own social distancing behaviour. The reciprocal relationship between social identity and group norms is well acknowledged in contemporary models of identity formation (see Postmes, Haslam, & Swaab, 2005; Smith et al., 2015), but the moderating influence of individual behaviour in this relationship has not been modelled explicitly; behaviour is typically considered a *consequence* of identity or norm formation. In contrast, the SIMBA presents a theoretical framework wherein individual behaviour also has explanatory power in the prediction of identity‐related constructs. Our findings emphasize that behaviours, such as social distancing, need not be thought of solely as an outcome to be predicted by other variables. While having significant direct consequences for health and disease spread, behavioural engagement in protective health behaviour also has additional implications for the comprehension and prediction of one's social identity and their perception of what is normative for a particular group membership.

Although we expected associations to fluctuate in strength during the early months of the pandemic, this was only the case for the group norm (i.e. group‐behaviour) associations and the explicit behaviour (i.e. self‐behaviour) association. Most associations demonstrated stability over time. British national identification was particularly stable on both implicit and explicit measures, which is unsurprising given that it was an exceptionally strong association that, unlike the group norm and behaviour associations, was a pre‐existing association. Moreover, it is likely that this association was further strengthened and validated during the heights of the pandemic due to the increased salience of national identities within this context (Bieber, 2020)—whereby protective health behaviours were essential not only for the sake of the self, but also for the community as a whole.

In contrast, the group norm and behaviour associations demonstrated less stability. The group norm association—which was less stable on both implicit and explicit measures^15^—represented a recently emerging association that likely captured fluctuations in public behavioural engagement (Wright et al., 2021) and a perceived lack of clarity surrounding government guidelines (Williams et al., 2020, 2021). Indeed, implicit measures have been found to show particularly low levels of temporal stability when changes in the broader context activate different associations at different measurement points (see Gschwendner et al., 2008; Rydell & Gawronski, 2009). Validation of propositional information that is consistent with recently activated associations could be responsible for convergence among implicit and explicit measures of the group‐behaviour relationship (see Gawronski & Bodenhausen, 2006, 2011). However, in the case of the self‐behaviour relationship, these measures diverged. The activated implicit association—which might be sensitive to the recurrent contextual reminders to socially distance—may have later been rejected as a basis for overt judgement when found to be inconsistent with other relevant information (e.g., reduced personal engagement in social distancing, media reports suggesting low levels of compliance). This would lead to lesser stability on explicit measures only.

Findings regarding the reciprocal, long‐range prediction of British national identity, group norms and social distancing behaviour were inconsistent with predictions. Balance among concepts was found at each timepoint, but each outcome variable at Time 2 was not explained by the interaction between the change scores (i.e. the change in associative strength between Time 1 and Time 2) of the two remaining variables. This could be attributed to the degree of stability demonstrated by most associations across timepoints; identity and social distancing behaviour were found to remain consistent irrespective of the changes in governmental guidance between during and post‐lockdown. Moreover, the majority of variance in the outcome variable at Time 2 was predicted by the same variable at Time 1—leaving little to be explained by the change scores. The only outcome variables found to be explained by anything other than their corresponding Time 1 measurement were explicit (VAS) group norms and social distancing behaviour—both associations that changed significantly over time. It is possible that longitudinal relations among self‐group‐behaviour concepts may only be demonstrated over longer time periods—where the influence of past behaviour may diminish—or where associative strength is lower in the first instance and more susceptible to change over time.

### Theoretical implications, limitations and future directions

Previous tests of the SIMBA have examined associations among student and national identities in relation to the health‐related behaviour of alcohol consumption (Hughes & Smith, 2024). The present research demonstrates the assumptions of the model to generalize beyond this outcome variable of interest—highlighting the potential of the SIMBA to be applied more broadly to the measurement of any normative behaviour that is group‐based and identity driven. Moreover, the current findings provide novel insights into the temporal stability of self‐group‐behaviour associations, and the balance that is assumed to exist among them. Given that research on balanced identity more generally has used cross‐sectional designs, this study is the first to demonstrate that identity‐based triads of associations are stable across time at both an implicit and explicit level. However, the present research examined stability over a relatively short timeframe. The second measurement timepoint of the present research was intentionally selected due to the salience of the change in governmental guidance (e.g. soon after social distancing restrictions were lifted) to examine the extent to which associative patterns may change, given the changing contextual landscape of the pandemic. Nevertheless, future tests of the model will benefit from measuring associations over a longer period to provide greater insight into the temporal stability of associations, and balance‐congruity (e.g. do interactions among identity and norms at an earlier timepoint predict later behaviour, and if so, for how long is this consistency sustained and influential in long‐term behavioural engagement?).

Previous research examining balanced identity via the theoretical framework of the SIMBA (e.g. Hughes & Smith, 2024) and BIT (e.g. Cvencek et al., 2012, 2021; Greenwald et al., 2002) has applied these frameworks to the measurement of strong, pre‐existing associations among concepts. In examining social distancing behaviour—the performance of which was uncommon prior to March 2020—in relation to the British national identity, we demonstrate that the assumptions of the SIMBA do not apply solely to the measurement of associations that are supported by a long history of gradual revision and repeated activation. Rather, the current research highlights the value of the SIMBA in its ability to reflect reciprocal relations among novel real‐world associations as they emerge. It also supports the predictions of the model in relation to the initial, emergent form that group‐level cognitions take, suggesting that new cognitions are constrained by existing ones. Accordingly, balanced relations among social identity, group norms and behaviour appear to be emergent phenomena that need not rely on prolonged, iterative processes of enculturation or associative reinforcement. The design of the present research provides stronger evidence for this supposition than could be ascertained from previous tests of the SIMBA (Hughes & Smith, 2024). However, experimental tests, as opposed to the correlational tests reported here, are needed to directly speak to the issue of causality in the emergence of balance‐congruity and to establish the SIMBA as both a descriptive *and* a predictive model of group‐based behaviour.

### Practical implications

Although the primary aim of our research was to provide a further test of the utility and validity of the SIMBA in the context of the pandemic, our research also offers practical suggestions for communicators and policymakers—given that compliance with social distancing can have consequences for the trajectory of a pandemic. Our findings reiterate that creating, facilitating and communicating clear group norms has implications for widespread adherence to public health behaviours, such as social distancing. Hence, we echo recent research in this area (e.g. Bonell et al., 2020; Neville et al., 2021; Ruggeri et al., 2024; Ryoo & Kim, 2021; van Bavel et al., 2020) by recommending that efforts to achieve behaviour change should focus on correcting normative misperceptions. However, our findings also highlight that norms can be conceptualized, and influence behaviour, both implicitly and explicitly. Norm‐based interventions are typically implemented at the explicit level (e.g. through the provision of normative information; Lewis & Neighbors, 2006; Neighbors et al., 2004; Schultz, 1999), but altering associations in this way may not always be sufficient to induce downstream change at an implicit level (Hu et al., 2017)—where associations are deep‐rooted and learned via associative processes. Our research emphasizes the importance of targeting norms not only explicitly but also implicitly (e.g. through directly manipulating implicit group‐behaviour associations) to ensure that changes in behaviour are enduring.

Our findings also emphasize the interactive relationship between group norms and social identity; that is, group norms are most predictive of behaviour for those who identify highly with the group. Consequently, group norms surrounding social distancing behaviour are likely to be most influential when tied to shared social identities (Abrams et al., 1990), as is the case for the spread of other health behaviours (Centola, 2011). Messages that provide inclusive in‐group references for norms (e.g. members of your community, country, nation) may be most effective—particularly if communicators place themselves as members of the in‐group (Haslam, 2020) and frame normative messaging in terms of who ‘we’ are and how ‘we’ behave (Bonell et al., 2020). Over and above replicating the identity by norm interaction on self‐report measures, we are the first to find evidence of this interaction on implicit measures. Consequently, it is also important that communication facilitates the *implicit* link between the self and group, and group and behaviour; repeated verbal information, for example, has been found to have associative effects (Gawronski & Bodenhausen, 2006; Rydell & McConnell, 2006). Indeed, speeches from leaders most highly commended for encouraging behaviour change during the COVID‐19 pandemic make the most frequent use of collective pronouns (Haslam, 2020). Communication efforts that aim to achieve widespread behaviour change will benefit from recognizing that normative influence occurs *through* shared social identification at both an implicit and explicit level.

### Concluding remarks

In sum, through investigating associations among British national identity, group norms and social distancing behaviour in the context of COVID‐19, the current research highlights the utility of the SIMBA as a novel means of capturing relations among newly emergent self‐group‐behaviour concepts. Overall, these associations were found to be relatively stable and consistently demonstrated balanced configurations both during and post‐lockdown. However, the strength of any one association, as measured post‐lockdown, was not predicted by the interaction between the change scores of the remaining two. Future research could investigate longitudinal relationships over longer periods of time—where associative strength may show greater fluctuation, and the influence of past behaviour is more likely to wane. While bolstering the theoretical assumptions of the SIMBA in providing evidence of cognitive balance at both an implicit and explicit level, the current research sheds light on some of the key social psychological determinants of protective public health behaviour. When faced with future health public health crises that demand mass behaviour change, appreciating the mutual influence of social identification and group norms will be crucial to ensure the effective and timely modification of group‐based behaviour.

## AUTHOR CONTRIBUTIONS

**Emily A. Hughes:** Conceptualization; methodology; data curation; software; investigation; validation; formal analysis; funding acquisition; visualization; project administration; resources; writing – original draft; writing – review and editing. **Joanne R. Smith:** Conceptualization; methodology; validation; supervision; funding acquisition; project administration; resources; writing – review and editing.

## CONFLICT OF INTEREST STATEMENT

The authors declare no conflict of interest.

## Supporting information


Data S1.

## Data Availability

The data that support the findings of this study are openly available under the ‘Data & Analysis’ section of the project Open Science Framework (OSF) page at https://osf.io/ut6w7/. Data citation: [data set] Hughes, E. A., & Smith, J. R. 2021. Study_covid_sav. Open Science Framework. https://osf.io/ut6w7/.

## Footnotes

^1^
The SIMBA's appeal to associations draws on a long‐established ‘theory‐uncommitted’ understanding of the term that implies nothing more complex than a (physiologically unspecified) link that can allow one mental concept to activate another.

^2^
To avoid engaging in debate regarding whether our chosen measurement outcome (e.g. the Implicit Association Test) is *truly* implicit, we follow the terminological suggestions of De Houwer (2006). Accordingly, implicit measures are defined in relation to the *functional* properties of the outcome of the measurement procedure, rather than the *objective* properties of the measurement procedure itself. A conditional approach to the term ‘implicit’ is taken, whereby our measures can be considered to be ‘automatic’ in the sense that—relative to ‘explicit’ measures—participants are less aware of the outcome being measured (see Monteith et al., 2001) and have less control over the resultant effect (see Steffens, 2004). We do not make claims, nor engage in debates, regarding the superiority of either measurement type (e.g. whether implicit measures are ‘truer’ or ‘more accurate’). Rather, we are interested in comparing automatic and deliberative processing (see Fazio & Olson, 2003) from a balanced identity perspective.

^3^
Two additional hypotheses were pre‐registered, which aimed to compare the well‐being of those demonstrating cognitive consistency (i.e. balance) and cognitive inconsistency (i.e. imbalance) in their associations at both Time 1 and Time 2. However, the overwhelming majority of participants displayed balanced triads of associations, and therefore it was not possible to conduct the analyses pertaining to these hypotheses.

^4^
H2 is non‐directional, and following the balance‐congruity principle, the direction of each outcome (e.g. whether it is of positive or negative associative strength) specified in H4 through H7 is dependent on the nature of the interaction between the remaining two predictor variables. When predicting individual behaviour, for example, if an individual identifies as British (positive association) and perceives distancing behaviour to be normative for this group (positive association), then they will perceive themselves as engaging in distancing behaviour (positive association). However, if this same individual did not perceive distancing to be normative (negative association), then to maintain balance‐congruity, they would not perceive themselves as engaging in distancing behaviour (negative association). A direction is explicitly stated for all other hypotheses.

^5^
H7 (i.e. the longitudinal prediction of outcome variables at Time 2) represents a deviation from our pre‐registered hypotheses, as our pre‐registered longitudinal hypothesis was framed in a way that required it to be analysed and interpreted via the four‐test method (see Data S1). However, this would not have been a valid analysis to perform, given that it would not have allowed us to control for the influence of the outcome variables at Time 1.

^6^
A target sample size of 150 at Time 1 was based on the sample collected in previous tests of the SIMBA investigating similar constructs in both online and lab environments (Hughes & Smith, 2024; *N* = 120), acknowledging that sample size may closer resemble this at Time 2 following attrition. Target sample size also reflects the average sample size typically collected in tests of balanced identity (see Cvencek et al., 2012). Sample size was determined before any data analysis.

^7^
Data collection at Time 2 was terminated once no new responses had been collected for 3 weeks.

^8^
Counterbalancing of IATs was not possible, as IATs were programmed using IATGEN (Carpenter et al., 2019). This required each IAT to be integrated within Qualtrics as a separate survey, linked by URL.

^9^
Explicit measures of well‐being—including negative affect (PANAS; Watson et al., 1988), mental well‐being (WHO‐5; Staehr, 1998) and judgemental well‐being (SWLS; Diener et al., 1985)—were also collected, though not central to the hypotheses reported within the manuscript due to the deviation from pre‐registration reported in Footnote 3; see study pre‐registration for full details of these measures.

^10^
Detailed elaboration of the analysis strategy can be found in both the study pre‐registration and Data S1.

^11^
The within‐study meta‐analysis represents a departure from our pre‐registered data analysis plan. The pre‐registration specifies that data will be analysed and interpreted using the four‐test method (see Greenwald et al., 2002). Historically, passing all four tests of the four‐test method has been used as a criterion for theoretical confirmation of the balance‐congruity principle, and the first author was not aware of alternatives at the time of pre‐registration. However, this strategy has several caveats, the most notable being that its criterion are relatively conservative and that it does not provide a quantitative indicator of the magnitude of confirmation with regards to the balance‐congruity principle. Evidence for the superiority of the within‐study meta‐analysis has since been published (see Cvencek et al., 2021) with open access to code for reproduction (see Cvencek et al., 2020). An interpretation of results from the two‐step moderated regressions pertaining to the four‐test method, and accompanying simple slopes analyses, are nevertheless reported in Data S1. The pattern of results is substantively similar across the two methods.

^12^
As H7 represents a deviation from our pre‐registered hypotheses (see Footnote 5), analyses pertaining to H7 also represent a deviation from our pre‐registered data analysis strategy.

^13^
Absolute values ≥.65 would indicate a ‘strong’ association, values ≥.35 a ‘moderate’ association and values ≥.15 a ‘slight’ association.

^14^
Analyses find that all outcome measures can be exchanged in the same multiplicative assumption of the balance‐congruity principle. Results from the two‐step moderated regressions pertaining to the four‐test method support this interpretation, and are reported separately for each outcome variable (i.e. social identity, group norms and individual‐level behaviour) in Data S1.

^15^
Although important to note that this effect was only ‘small’ (Cohen, 1988).
