# Supplementary material for: Measuring associations among British national identification, group norms and social distancing behaviour during the COVID‐19 pandemic: Testing a Social Identity Model of Behavioural Associations (SIMBA)
Source: Br J Soc Psychol. 2025 Mar 21;64(2):e12862. doi: 10.1111/bjso.12862 (PMC11927383; doi:10.1111/bjso.12862)
Supplement: Supplementary file 1 — Data S1. [file BJSO-64-0-s001.docx]

**Supplemental Material: The Four-Test Method**

In accordance with the four-test method (Greenwald et al., 2002), four expectations are to be appraised from the two-step regression when determining support for the balance-congruity principle: (a) the two-predictor product at Step 1 should be statistically significant and possess a numerically positive standardised regression coefficient; (b) the interaction term’s regression coefficient should remain numerically positive at Step 2; (c) the increment in variance explained at Step 2 following the addition of the two individual predictors should not be statistically significant; and (d) the standardised regression coefficients associated with the two individual predictors at Step 2 should also not be statistically significant. Thus, 12 tests should be passed—four tests for each of the three criteria. A failure at step (a) indicates that the primary prediction of the theory (i.e., a basic fit of a multiplicative theoretical model) has not been confirmed.

**Testing the SIMBA: Time 1 Balanced Identity Analyses**

Table 1 provides a summary of the Time 1 balanced identity analyses for all implicit and explicit data. Overall, evidence for balance was strong at the implicit level, with all 12 possible tests passed—demonstrating that each measure’s prediction by the other two conformed to the pure multiplicative pattern. At the explicit level, the zero-point VAS measures provided greater support for balance than the Likert scale measures; 11 of the possible 12 tests were passed on the VAS’, compared to only seven on the scale measures. On explicit Likert scale measures, there was an overall trend of the interaction term being significant at both steps of the regression, in addition to one of the main effects entered at Step 2; each criterion appeared not to be a *sole* multiplicative product of the two remaining measures.

Table 1



*Summary of Time 1 Balanced Identity Analyses for all Implicit and Explicit Measures*

|  |  | Regression step | | | |  |  |  | Total tests passed |
| --- | --- | --- | --- | --- | --- | --- | --- | --- | --- |
|  | | Step 1 | |  | Step 2 | | | |  |
|  | Criterion | Interaction^a^ | *R^2 a^* |  | Interaction^b^ | *R^2^*_ch_ ^c^ | Direct effect 1^d^ | Direct effect 2^d^ |  |
| Implicit | Behaviour | **.40** | **.16***** |  | **.72** | **.01** | **-.18** | **-.23** | 12/12 |
|  | Identity | **.40** | **.16***** |  | **.15** | **.02** | **.22** | **.12** |  |
|  | Norms | **.42** | **.17***** |  | **.64** | **.02** | **-.25** | **-.01** |  |
| Explicit (VAS) | Behaviour | **.26** | **.07** |  | **.43** | **.02** | **-.08** | **-.12** | 11/12 |
|  | Identity | **.42** | **.18***** |  | **.50** | **.002** | **-.08** | **-.02** |  |
|  | Norms | **.43** | **.18***** |  | **1.21** | **.02** | -.54* | **-.60** |  |
| Explicit (Scale) | Behaviour | .13 | .02 |  | **.17** | **.02** | **.07** | **.10** | 7/12 |
|  | Identity | **.24** | **.06**** |  | **.19** | .15*** | .39*** | **-.02** |  |
|  | Norms | **.19** | **.04*** |  | **.14** | .16*** | **.06** | .39*** |  |

*Notes.* Alpha levels are * *p*<.05, ** *p*<.01, *** *p*<.001.

Cells in bold represent results consistent with predictions of the SIMBA.

Direct effect 1 = identity and direct effect 2 = norms where criterion is behaviour.

Direct effect 1 = behaviour and direct effect 2 = norms where criterion is identity.

Direct effect 1 = behaviour and direct effect 2 = identity where the criterion is norms.

^a^ Interaction term should be statistically significant and regression coefficient positive in order to past test.

^b^ Should remain numerically positive in order to pass test.

^c^ Should not be statistically significant in order to pass test.

^d^ Should both not differ statistically from zero in order to pass test.

**Testing the SIMBA: Time 2 Balanced Identity Analyses**

Table 2 provides a summary of the Time 2 balanced identity analyses for all implicit and explicit data. Overall, evidence for balance was strong at the implicit level, and also at the explicit level regarding data from VAS’. On both these measures, all 12 possible tests were passed—demonstrating that each measure’s prediction by the other two conformed to the pure multiplicative pattern. However, only six of the possible 12 tests were passed on the explicit Likert scale measures; the interaction term was consistently significant at both steps of the regression, in addition to at least one of the main effects entered at Step 2. Therefore, each criterion appeared not to be a *sole* multiplicative product of the two remaining measures.

Table 2



*Summary of Time 2 Balanced Identity Analyses for all Implicit and Explicit Measures*

|  |  | Regression step | | | |  |  |  | Total tests passed |
| --- | --- | --- | --- | --- | --- | --- | --- | --- | --- |
|  | | Step 1 | |  | Step 2 | | | |  |
|  | Criterion | Interaction^a^ | *R^2 a^* |  | Interaction^b^ | *R^2^*_ch_ ^c^ | Direct effect 1^d^ | Direct effect 2^d^ |  |
| Implicit | Behaviour | **.39** | **.15***** |  | **.37** | **.01** | **-.08** | **.08** | 12/12 |
|  | Identity | **.28** | **.08**** |  | **.15** | **.01** | **.16** | **.02** |  |
|  | Norms | **.41** | **.17***** |  | **.17** | **.01** | **.19** | **.15** |  |
| Explicit (VAS) | Behaviour | **.28** | **.08**** |  | **.41** | **.01** | **-.02** | **-.14** | 12/12 |
|  | Identity | **.46** | **.21***** |  | **.40** | **.01** | **.08** | **-.09** |  |
|  | Norms | **.48** | **.23***** |  | **.57** | **.02** | **-.17** | **.01** |  |
| Explicit (Scale) | Behaviour | **.18** | **.03*** |  | **.26** | .07** | **-.001** | .28** | 6/12 |
|  | Identity | **.27** | **.07**** |  | **.19** | .20*** | .46*** | **-.07** |  |
|  | Norms | **.31** | **.09***** |  | **.20** | .21*** | .15* | .44*** |  |

*Notes:* Alpha levels are * *p*<.05, ** *p*<.01, *** *p*<.001.

Cells in bold represent results consistent with predictions of the SIMBA.

Direct effect 1 = identity and direct effect 2 = norms where criterion is behaviour.

Direct effect 1 = behaviour and direct effect 2 = norms where criterion is identity.

Direct effect 1 = behaviour and direct effect 2 = identity where the criterion is norms.

^a^ Interaction term should be statistically significant and regression coefficient positive in order to past test.

^b^ Should remain numerically positive in order to pass test.

^c^ Should not be statistically significant in order to pass test.

^d^ Should both not differ statistically from zero in order to pass test.

**Supplemental Material: Pre-Registered Data Analysis Strategy**

**Statistical Models^[[1]](#footnote-1)^**

To test H1 investigating the presence of associations, two-tailed, one-sample t-tests will be conducted on the *D*-scores from each IAT to determine whether the *D*-score means are significantly different from zero. A positive *D*-score indicates an association between compatible target and attribute categories, such as ‘self’ and ‘British’.

To test H2, and establish whether associations strengthen or weaken between time one and time two, a two-tailed paired samples t-test will be conducted for the identity, group norms, and behaviour IATs (implicit), VAS (explicit), and Likert scales (explicit)—with the continuous DV of the relevant *D*-score/VAS/Likert scale score being measured at time one and time two in the same sample.

To test H3, a linear regression will be conducted, with post-lockdown behavioural intentions at time one predicting actual self-reported post-lockdown behaviour at time two.

For H4 through H6, and to test the balance-congruity principle, a series of moderated regressions will be conducted for both explicit and implicit measures at each timepoint. The traditional four-test method (Greenwald et al., 2002) will be employed, where each of the three implicit or explicit measures (identity, group norms and behaviour) serves as a criterion to be predicted by the remaining two. In step one, the criterion will be predicted solely by the multiplicative product of the other two measures. In step two, the two measures will be added individually to the regression as predictors. The typical order of estimating main effects before interaction effects must be reversed to determine whether the data of the balanced identity design can be fit entirely by the interaction term. To plot the interactions and determine whether the x-y relationship between constructs is significant at moderator values one standard deviation above and below the mean, simple slopes analyses will be conducted.

For both the implicit and explicit measures, correlational analyses will be performed in order to establish whether a relationship exists between the measures, and also whether the two methods produce similar outcomes. Zero-order correlations will also aid in descriptively supporting the extent to which the strength of any one association can be predicted by the combined strength of the remaining two (i.e., the balance-congruity principle).

**Transformations**

The derived explicit scale measures of group norms, behaviour, and social identification will be centred for all regressions and simple slopes. This will involve subtracting the exact mean from our original indices. Implicit measures will be centred for the reporting of simple slopes analyses. Implicit measures (in the main balance-congruity moderated regressions) and explicit VAS will not be centred under the assumption that they have a rational zero point.

**Inference Criteria**

We will use the standard criteria of *p* being less than 0.05 for all test results to determine statistical significance.

**Data Exclusion**

All participants will meet the inclusion criteria of British (English) nationality before entering the survey as a result of internal pre-screeners within Prolific. Participant data will be excluded if Prolific ID is not entered consistently and correctly before each IAT, and therefore, responses cannot be matched across tasks and/or timepoints. Responses of this sort will not be approved to receive remuneration. For the analysis of hypotheses that span timepoints, data will only be used for those completing both parts of the study.

**Supplemental Material: Simple Slopes Analyses**

**Time 1 Simple Slopes Analyses**

Simple slopes analyses on both implicit and explicit measures (see Table 1) were largely consistent with expectations; regarding the prediction of social distancing behaviour and group norms, the x-y relationships between constructs were only significant at high values of the moderator. Individuals displayed strong self-distancing associations (i.e., reported greater engagement in social distancing behaviour) when they perceived the British group norm to be in support of social distancing, but only when possessing a strong self-group association (i.e., identifying highly as British). Similarly, individuals displayed strong group-behaviour associations (i.e., perceived British norms in support of social distancing behaviour) when possessing strong self-group associations, but only if they also demonstrated strong self-distancing associations themselves (i.e., reported increased social distancing behaviour). However, the prediction of implicit and explicit social identity was less consistent with expectations; the norm-identity relationship was significant for those demonstrating both strong and weak self-distancing associations (i.e., for those engaging in social distancing behaviour at both high and low levels)—despite the effect being stronger for those displaying associations of greater strength (i.e., engaging at high levels).

Table 1

*Implicit and Explicit Simple Slopes Analyses (Time 1)*

|  |  |  | Moderator values | | | | | | |
| --- | --- | --- | --- | --- | --- | --- | --- | --- | --- |
|  |  |  | High (+1 SD) | | |  | Low (-1 SD) | | |
|  | Criterion | Moderator | β | 95% CI | *P* |  | β | 95% CI | *p* |
| Implicit | Self-Distancing | Self-Group | **.40** | **[.199, .726]** | **<.001** |  | **.03** | **[-.222, .293]** | **.788** |
|  | Self-Group | Self-Distancing | **.33** | **[.110, .640]** | **.006** |  | .25 | [-.004, .566] | .053 |
|  | Group-Distancing | Self-Distancing | **.50** | **[.218, .654]** | **<.001** |  | **.10** | **[-.116, .299]** | **.385** |
| Explicit (VAS) | Behaviour | Identity | **.40** | **[.104, .357]** | **<.001** |  | **-.01** | **[-.141, .130]** | **.937** |
|  | Identity | Behaviour | **.55** | **[.267, .570]** | **<.001** |  | .23 | [.025, .320] | .022 |
|  | Norms | Behaviour | **.64** | **[.513, 1.150]** | **<.001** |  | **.01** | **[-.392, .404]** | **.976** |

*Notes.* Cells in bold represent results consistent with predictions of the SIMBA.

Due to a lack of support for balance-congruity on explicit (scale) measures, simple slopes analyses are presented only for implicit and explicit (VAS) data.

**Time 2 Simple Slopes Analyses**

Simple slopes analyses regarding the prediction of group norms, both implicitly and explicitly, were consistent with expectations (see Table 2); the identity-norm relationship was only significant at high values of the moderator (i.e., social distancing behaviour). Accordingly, individuals displayed strong group-behaviour associations (i.e., perceived British norms in support of social distancing behaviour) when possessing strong self-group associations, but only if they also demonstrated strong self-distancing associations themselves (i.e., reported increased social distancing behaviour).

Analyses regarding the prediction of implicit social identity, and explicit social distancing behaviour, were also consistent with expectations. Implicitly, individuals demonstrated stronger self-group associations when associating the British identity with social distancing behaviour, but only if they showed strong self-distancing associations themselves. Explicitly, individuals reported greater engagement in social distancing behaviour when they perceived a British group norm in support of distancing, but only if they strongly identified as British themselves. However, analyses regarding the prediction of explicit social identity, and implicit social distancing behaviour, were less consistent with expectations; here, the norm-identity and norm-behaviour relationships were significant at both high and low values of the moderator—despite the effect being stronger at high values.

Table 2

*Implicit and Explicit Simple Slopes Analyses (Time 2)*

|  |  |  | Moderator values | | | | | | |
| --- | --- | --- | --- | --- | --- | --- | --- | --- | --- |
|  |  |  | High (+1 SD) | | |  | Low (-1 SD) | | |
|  | Criterion | Moderator | β | 95% CI | *P* |  | β | 95% CI | *p* |
| Implicit | Self-Distancing | Self-Group | **.48** | **[.210, .601]** | **<.001** |  | .24 | [.045, .371] | .013 |
|  | Self-Group | Self-Distancing | **.29** | **[.051, .480]** | **.016** |  | **.19** | **[-.050, .398]** | **.127** |
|  | Group-Distancing | Self-Distancing | **.28** | **[.037, .577]** | **.026** |  | **.18** | **[-.043, .433]** | **.108** |
| Explicit (VAS) | Behaviour | Identity | **.42** | **[.123, .441]** | **<.001** |  | **-.004** | **[-.186, .182]** | **.979** |
|  | Identity | Behaviour | **.59** | **[.309, .645]** | **<.001** |  | .26 | [.019, .404] | .031 |
|  | Norms | Behaviour | **.60** | **[.462, 1.014]** | **<.001** |  | **.22** | **[-.030, .580]** | **.077** |

*Notes.* Cells in bold represent results consistent with predictions of the SIMBA.

Due to a lack of support for balance-congruity on explicit (scale) measures, simple slopes analyses are presented only for implicit and explicit (VAS) data.

1. Pre-registered analyses that are not reported in the manuscript’s main text (see Footnotes 3 and 5 of the main text) are omitted from this reporting. To present the hypotheses in a more concise and logical manner in the main text, we deviated from the numbering system adopted in the pre-registration. We have updated the numbering of hypotheses here so that it aligns with the main text. [↑](#footnote-ref-1)
